# A preliminary randomised controlled study of short-term *Antrodia cinnamomea* treatment combined with chemotherapy for patients with advanced cancer

**DOI:** 10.1186/s12906-016-1312-9

**Published:** 2016-08-26

**Authors:** Ming-Yen Tsai, Yu-Chiang Hung, Yen-Hao Chen, Yung-Hsiang Chen, Yu-Chuen Huang, Chao-Wei Kao, Yu-Li Su, Hsien-Hsueh Elley Chiu, Kun-Ming Rau

**Affiliations:** 1 Department of Chinese Medicine, Kaohsiung Chang Gung Memorial Hospital and Chang Gung University College of Medicine, Kaohsiung, 83301 Taiwan; 2 Graduate Institute of Integrated Medicine, College of Chinese Medicine, China Medical University, Taichung, 40402 Taiwan; 3 School of Chinese Medicine for Post Baccalaureate, I-Shou University, Kaohsiung, 83301 Taiwan; 4 Department of Internal Medicine, Division of Oncology, Kaohsiung Chang Gung Memorial Hospital and Chang Gung University College of Medicine, Kaohsiung, 83301 Taiwan; 5 Department of Medical Research, China Medical University Hospital and School of Chinese Medicine, China Medical University, Taichung, 40402 Taiwan

**Keywords:** Antrodia cinnamomea, Chemotherapy, Advanced and metastatic cancer, Outcome

## Abstract

**Background:**

*Antrodia cinnamomea* (AC) is a popular medicinal mushroom in Taiwan that has been widely used for treatment of various cancers. Few clinical studies have reported its application and efficiency in therapeutic chemotherapy strategies. We performed a double-blind, randomized clinical study to investigate whether AC given for 30 days had acceptable safety and efficacy in advanced cancer patients receiving chemotherapy.

**Methods:**

Patients with advanced and/or metastatic adenocarcinoma, performance status (PS) 0–2, and adequate organ function who had previously been treated with standard chemotherapy were randomly assigned to receive routine chemotherapy regimens with AC (20 ml twice daily) orally for 30 days or placebo. The primary endpoint was 6-month overall survival (OS); the secondary endpoints were disease control rate (DCR), quality of life (QoL), adverse event (AE), and biochemical features within 30 days of treatment.

**Results:**

From August 2010 to July 2012, 37 subjects with gastric, lung, liver, breast, and colorectal cancer (17 in the AC group, 20 in the placebo group) were enrolled in the study. Disease progression was the primary cause of death in 4 (33.3 %) AC and 8 (66.7 %) placebo recipients. Mean OSs were 5.4 months for the AC group and 5.0 months for the placebo group (*p* = 0.340), and the DCRs were 41.2 and 55 %, respectively (*p* = 0.33). Most hematologic, liver, or kidney functions did not differ significantly between the two groups, but platelet counts were lower in the AC group than in the placebo group (*p* = 0.02). QoL assessments were similar in the two groups, except that the AC group showed significant improvements in quality of sleep (*p* = 0.04).

**Conclusions:**

Although we found a lower mortality rate and longer mean OS in the AC group than in the control group, *A. cinnamomea* combined with chemotherapy was not shown to improve the outcome of advanced cancer patients, possibly due to the small sample size. In fact, the combination may present a potential risk of lowered platelet counts. Adequately powered clinical trials will be necessary to address this question.

**Trial registration:**

ClinicalTrials.gov NCT01287286.

## Background

Cancer is a serious health problem in Asia, and it is now the leading cause of death in Asian Pacific countries such as China, Japan, Korea, and Taiwan. The most commonly diagnosed cancers in Eastern Asia, in descending order, are lung, breast, colon, and rectum, and the leading causes of cancer deaths are lung, stomach, and liver cancer [1]. Garcia et al. reported that in the year 2007, an estimated 3.3 million people were diagnosed with cancer and 2.3 million people died from cancer in Eastern Asia. Their report also predicted that by 2020, an estimated 7.1 million new cases of cancer will be diagnosed each year if existing prevention and management strategies remain unchanged [2].

The major conventional therapies for cancer are surgery, chemotherapy, and radiotherapy. However, these therapies have numerous limitations: (1) Most cancer patients are diagnosed too late to undergo surgery, despite advances in biomarker and radiographic research; (2) although chemotherapy and radiotherapy are effective against cancer, they also have serious side effects and complications (e.g., fatigue, pain, diarrhea, nausea, vomiting, and skin reactions); (3) most advanced cancers have a survival rate of less than 5 years, and recurrence or metastasis is quite common even after surgical resection; and (4) since some cancers are relatively chemo- or radio-resistant, systemic cytotoxic chemotherapy and radiotherapy are minimally effective at improving patient survival [3, 4]. For these reasons, and in spite of the uncertainty of its benefits due the lack of well-controlled scientific studies, cancer patients have increasingly turned to complementary and alternative medicine (CAM) for treatment [5]. A recent study showed that in Taiwan, up to 50.4 % of adults and 84.6 % of females use some form of CAM to treat cancer [6].

*Antrodia cinnamomea* (AC), a well-known medical mushroom used in Taiwan, has several pharmacological functions, including antioxidant, anti-itching and hepatoprotective effects, as demonstrated by experimental studies [7, 8]. AC extract contains a complex mixture of bioactive ingredients, including triterpenoids, steroids, polysaccharides, and phenyl and biphenyl compounds [9]. It has recently become popular as a potential complementary and alternative therapeutic agent for the treatment of various cancers [10]. Previous studies revealed that maleimide derivative isolated from AC attenuates the migration and invasion of breast cancer cells [11]. Investigations by Hsu et al. have shown that AC fruiting body extract exhibits a significant cytotoxic effect against hepatoma cell line Hep G2 and PLC/PRF/5 cells [12]. Furthermore, AC extract has adjuvant antiproliferative effects on hepatoma cells in vitro and in vivo when combined with anti-tumor agents through the inhibition of Multi-Drug Resistance (MDR) gene expressions and the pathway of COX-2-dependent inhibition of phospho-AKT (p-AKT) [13]. However, the clinical effects of AC on cancer have not yet been demonstrated in human trials. To increase understanding of the effects of *A. cinnamomea* on cancer patients, we designed the preliminary study and worked with oncologists and a manufacturer to settle some of the controversy surrounding AC treatment. In addition, we also evaluated the safety and the alleviation of symptoms as a result of treatment with AC in combination with chemotherapy for patients in advanced stages.

## Methods

### Study design and patient selection

This prospective randomized, double-blind study was undertaken in the Medical Oncology Department of Kaohsiung Chang Gung Medical Hospital (KCGMH) between August 2010 and July 2012. The protocol was approved by the KCGMH Ethics Committee (IRB-98-3904A3) and registered at clinicaltrials.gov under the identification code NCT01287286. All participants provided written informed consent to participate in the study.

Patients aged ≥18 years were eligible if they had advanced or recurrent, untreated, stage III-IV adenocarcinomas that were histologically or cytologically confirmed; had previously received standard chemotherapy regimens; and had an Eastern Co-operative Oncology Group (ECOG) performance status (PS) of 0–2. Patients were excluded if they were pregnant or lactating; had used any other investigational agent within the 28 days prior to registration; had a severe current illness; had an abnormal blood cell count, liver function test, or creatinine clearance; or had known hypersensitivity to any formulas of AC in the market.

### Treatment

Eligible patients were randomly assigned to either the AC group or the placebo group. The participants were given 20-ml oral formulations twice a day for 30 days. The *A. cinnamomea* used in the study comprised mainly 2100 mg polysaccharides, 172 mg triterpenoids, and 2687.5 mg γ-aminobutyric acid, and its Taiwan Symbol of National Quality (SNQ) approval number was A00851. The aqueous extract from mycelium of AC displayed cytotoxic activity with an IC50 value > 400 μg/ml in murine RAW 264.7 cells. In addition, the freeze-dried state of AC mycelia was administered for 28 days and 90 days respectively to evaluate the oral toxicity in Sprague-Dawley (SD) rats [14, 15]. Both studies showed the no-observed-adverse-effect-level (NOAEL) and are safe to use as dietary supplements or nutraceuticals.

Strains of AC were provided by the Research and Development Center (New Bellus Enterprises Co., Ltd, Tainan, Taiwan). Extracts were prepared as previously described by Cheng et al [16]. Briefly, AC was first cultured with Malt Extract Agar (MEA) I broth containing 1.5 % agar, 1.5 % malt extract, 2 % glucose, and 0.1 % peptone at 30 °C for 7–10 days. AC mycelia were inoculated into MEA I broth and incubated at 30 °C with shaking for 8–10 days. After sterilization at 121 °C for 30 min, fermented extracts were centrifuged at 3000 g for 10 min and then permeated through a 3000 MW ultrafiltration membrane. The placebo was made of malt, sucrose, and xanthan gum to achieve comparable color, outlook, and taste to the AC. Both the AC and placebo solutions were produced by Amon Biotech Co., Ltd (Lot no. AC9905245TA, Expiry Date/ EXP.: June 2010/ June 2013). Quality control was ensured. The AC and placebo solutions were packed in sealed opaque aluminum packages; only the treatment codes and administration instructions were printed on the outsides of the package to ensure successful blinding of the patients.

Eligible patients with lung cancer were scheduled to receive platinum-based chemotherapy with cisplatin dosed at 60 − 75 mg/m^2^ or a carboplatin area under the curve (AUC5). The following regimens were sequentially adopted for this study at the discretion of the investigator in accordance with the National Comprehensive Cancer Network (NCCN) guidelines for patients with breast, stomach, liver, and colorectal cancers: adriamycin-based, taxane- or vinorelbine-based, 5-fluorouracil (5-FU)-based and oxaliplatin-based chemotherapy (Table 1) [17]. After the first dose of standard chemotherapy, the patients began treatment with a 30-day supply of the agent and were asked to return the containers at the next scheduled visit (on days 7, 15, and 30). Counting the unused boxes served as a measure for compliance with treatment. Patients were withdrawn from the trial if they failed to appear for the next two scheduled visits or if they had not used at least 80 % of the supplied treatment at 75 % of the treatment intervals.

**Table 1:** Chemotherapeutic protocols for different advanced cancer types according to NCCN guidelines and their adverse events

Cancer type	Chemotherapeutic regimens	Common adverse events
Lung	Platinum-based	Nausea, vomiting, nephrotoxicity, bone marrow suppression
Breast	Adriamycin-based or taxane-based	Bone marrow suppression, nausea, vomiting, alopecia
Stomach	Oxaliplatin/5-fluouracil	Nausea, vomiting, mucositis, diarrhea, bone marrow suppression
Liver	Adriamycin and Cisplatin	Nausea, vomiting, bone marrow suppression, nephrotoxicity
Colorectal	Oxaliplatin-based or Irinotecan-based	Nausea, vomiting, diarrhea, bone marrow suppression

### Efficacy and safety assessment

The primary endpoint of this analysis was overall survival (OS). OS was calculated from the date of randomization until the date of death or the last 6-month follow-up. The secondary endpoints were the disease control rate (DCR) (complete response (CR), partial response (PR), and stable disease (SD)), as determined by Response Evaluation Criteria in Solid Tumors (RECIST) version 1.1. Tumor assessments using computed tomography scans were performed before *A. cinnamomea* treatment and 30 days thereafter. Other secondary outcome measures included Quality of life (QoL), adverse event (AE), and clinical and laboratory assessments conducted at baseline and at 30 days after administration. QoL was measured using the European Organization for Research and Treatment-Quality of Life Questionnaire (EORTC-QLQ-C30) and the Multidimensional Fatigue Inventory (MFI-20). The incidence and severity of AEs were graded according to the National Cancer Institute’s Common Terminology Criteria for Adverse Events (CTCAE) version 3.0.

### Statistical analyses

The baseline characteristics and clinical data of the patients treated with chemotherapy plus AC or placebo are presented as number and mean ± standard deviation (SD). Overall survival (OS) for both groups was measured from randomization until death from any cause. Patients that were alive but lost to follow-up were excluded at the last follow-up date. OS was illustrated by means of Kaplan-Meier curves, and log-rank tests were used to compare survival between the two groups. The Chi-square test was used to compare the treatment response in the two groups, separated as DCR vs. progressive disease (PD). The data on the QoL, clinical, and laboratory assessments for the pre- and post-tests within the AC group and the placebo group were assessed by Wilcoxon signed-rank test. Differences in efficacy between the two groups were determined by Mann-Whitney *U*-test. All statistical analyses were performed with SPSS version 18 for Windows. A *p* value < 0.05 was considered statistically significant.

## Result

**Fig. 1:**
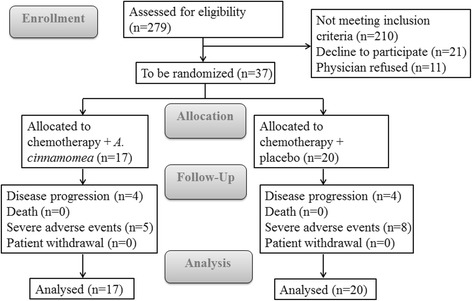
Flow chart

Between August 2010 and July 2012, a total of 37 patients were recruited into the study and considered for the full analysis (Fig. 1). Of the 37, 17 received chemotherapy with *A. cinnamomea*, and 20, chemotherapy with placebo. Patient demographics and disease characteristics are presented in Table 2.

**Table 2:** Baseline characteristics of patients treated with chemotherapy plus *A. cinnamomea* (AC) or placebo

	Chemotherapy + AC (*n* = 17)	Chemotherapy + placebo (*n* = 20)
Sex (male/female)	10/7	8/12
Age (Mean ± SD)	56.71 ± 9.61	53.10 ± 10.86
Body weight in kg (Mean ± SD)	61.98 ± 9.09	60.36 ± 11.11
Months of prior treatment (Mean ± SD)	9.80 ± 3.46	8.55 ± 4.03
Tumor location (TNM stage, AJCC, 2002)		
Lung (stage IV)	1	7
Breast (stage IV)	4	4
Stomach (stage III-IV)	5	5
Liver (stage III-IV)	3	1
Colorectal (stage IV)	4	3

**Fig. 2:**
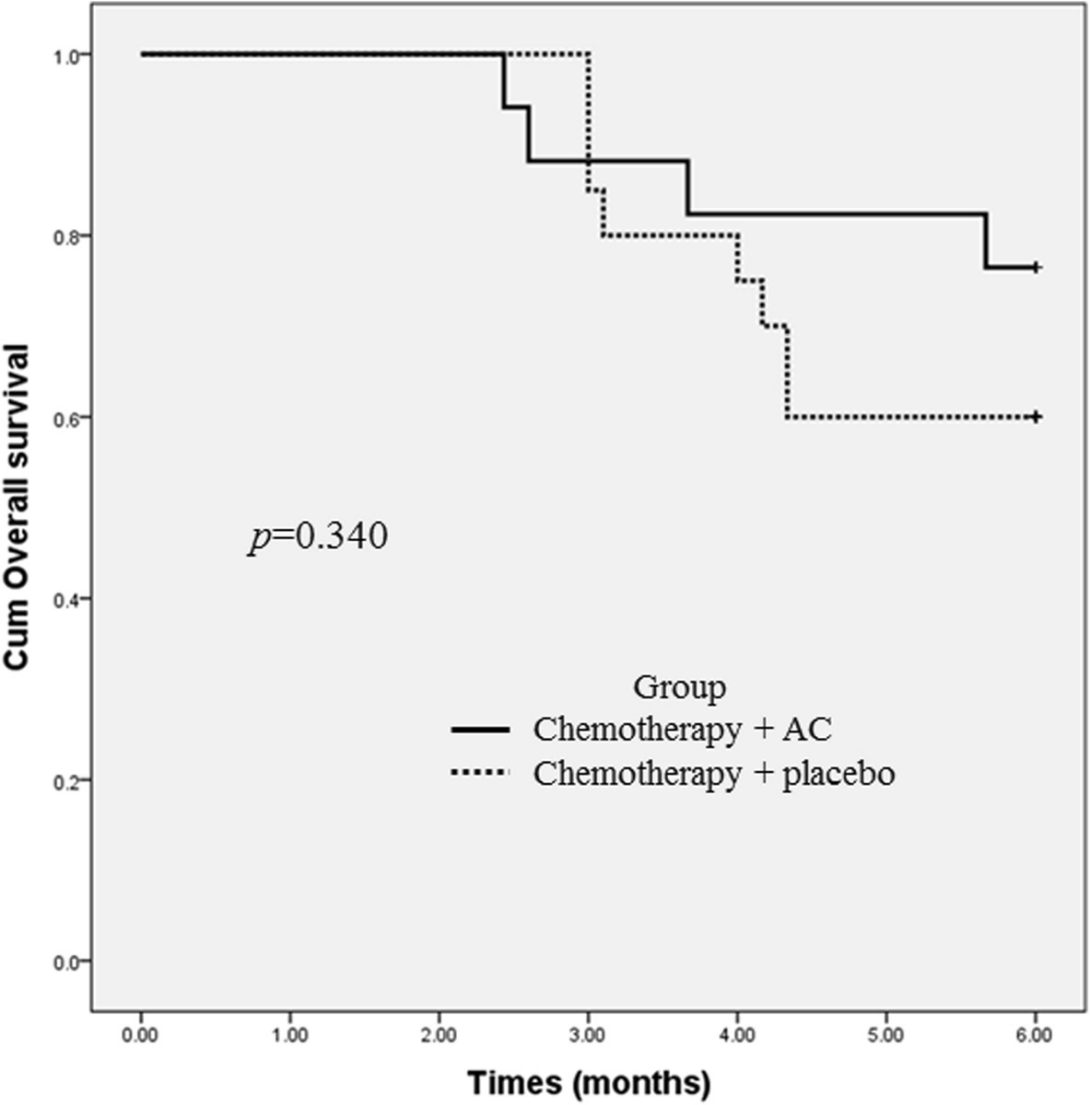
Kaplan-Meier curves of 6-month survival for chemotherapy plus *A. cinnamomea* (AC) or placebo

At the 30-day analysis, patients had experienced 2.35 and 1.75 chemotherapy cycles schedule while on AC and placebo treatment, respectively. Four cases of disease progression (23.5 %) had occurred in the AC group and 4 (20 %) in the placebo group, and no deaths or withdrawals were recorded. By the cut-off date at 6 months of treatment, a total of 12 patients had died (32.4 %), 25 patients were surviving (67.6 %), and 4 patients in the placebo group were lost to follow-up. Disease progression was the primary cause of death in 4 (33.3 %) AC and 8 (66.7 %) placebo recipients. There was no statistically significant difference in survival between the treatment groups; mean OSs were 5.4 months (AC group) and 5.0 months (placebo group; *p* = 0.340; Fig. 2). As shown in Table 3, 29 of the 37 patients (13 patients in the AC group and 16 patients in the placebo group) had measurable lesions and could be evaluated for response based on the RECIST criteria. No complete response was observed in either treatment group. Three patients in the AC group achieved PR, four patients were SD, and the remaining six patients were PD. In the placebo group, three patients achieved PR, eight patients were SD, and the remaining five patients were PD. The DCRs were 41.2 and 55 % in the AC and placebo groups, respectively. There were no significant differences in the objective DCR vs. PD in the two groups (*p* = 0.33).

**Table 3:** Best overall response and disease control rate per RECIST (Full Analysis Population)

	Chemotherapy + AC (*n* = 17)	Chemotherapy + placebo (*n* = 20)	*p*-value
Best overall response, No. (%)^a^			
Complete response (CR)	0	0	
Partial response (PR)	3 (17.6)	3 (15)	
Stable disease (SD)	4 (23.5)	8 (40)	
Progressive disease (PD)	6 (35.3)	5 (25)	
Unknown	4 (23.5)	4 (20)	
Disease control rate (CR + PR + SD)	7 (41.2)	11 (55)	0.33

^a^Proportion of patients whose best overall response is complete response, partial response, or stable disease according to RECIST (Response Evaluation Criteria in Solid Tumors)

All patients completed the two quality of life forms (Table 4). Most of the EORTC-QLQ-C30 assessments did not show significant differences between the treatment groups with regard to the mean scores from baseline to 30 days, but quality of sleep improved significantly more in the AC group than in the placebo group (*p* = 0.04). There was no difference in fatigue levels on the general subscale of the MFI-20 between the two groups.

**Table 4:** Quality of life of cancer patients treated with chemotherapy plus *A. cinnamomea* (AC) or placebo

	Chemotherapy + AC (*n* = 17)		*p*-value^a^	Chemotherapy + placebo (*n* = 20)		*p*-value^a^	*p*-value^b^
	Pre-test	Post-test		Pre-test	Post-test		
EORTCQLQ-C30							
General condition	49.51 ± 11.20	55.55 ± 28.63	0.08	53.75 ± 26.27	53.07 ± 23.10	0.28	0.76
Fatigue	26.79 ± 27.23	34.72 ± 36.03	0.18	28.33 ± 18.19	41.52 ± 28.16	0.06	0.32
Nausea and vomiting	22.54 ± 34.32	10 ± 18.68	0.08	9.16 ± 18.31	16.66 ± 26.64	0.24	0.51
Pain	14.70 ± 21.95	25.55 ± 30.12	0.04	19.16 ± 23.74	21.92 ± 28.89	0.38	0.77
Shortness of breath	9.80 ± 19.59	11.11 ± 20.57	0.31	13.33 ± 19.94	19.29 ± 25.61	0.15	0.30
Sleep disturbance	47.05 ± 42.58	40.00 ± 36.07	0.37	46.66 ± 38.08	61.40 ± 35.59	0.01	0.04
Lack of appetite	27.45 ± 35.81	22.22 ± 29.99	0.44	18.33 ± 27.51	31.57 ± 34.19	0.03	0.44
Constipation	15.68 ± 20.80	17.77 ± 27.79	0.28	21.66 ± 31.11	8.77 ± 18.73	0.02	0.29
Diarrhea	5.88 ± 13.09	6.66 ± 13.80	0.12	1.66 ± 7.45	10.52 ± 24.97	0.21	0.86
MFI - 20							
General Fatigue	10.41 ± 1.50	10.6 ± 2.26	0.35	10.55 ± 1.84	10.15 ± 2.03	0.21	0.41
Physical Fatigue	12.23 ± 0.90	12.13 ± 1.41	0.33	11.95 ± 0.82	12.25 ± 1.11	0.24	0.78
Reduced Activity	11.23 ± 1.60	11.66 ± 0.90	0.47	11.20 ± 1.54	11.15 ± 1.26	0.43	0.12
Reduced Motivation	12.76 ± 1.82	13.00 ± 1.41	0.15	12.55 ± 1.23	12.50 ± 1.39	0.33	0.37
Mental Fatigue	11.17 ± 1.07	11.33 ± 1.11	0.20	10.9 ± 1.77	11.95 ± 1.35	0.01	0.28

^a^Wilcoxon signed-rank test (Within group)

^b^Mann-Whitney *U* test (Differences in efficacy between two groups)

All laboratory parameters were comparable in the AC group and the placebo group as compared with baseline, and similar trends between both groups were observed throughout the study period (Table 5). After 30 days of treatment, the AC group showed a significant decrease in platelet count (*p* < 0.01). We also detected a significant difference in the decreases in the platelet counts in the two groups (*p* = 0.02). Other tested biomarkers did not demonstrate significant changes in the two groups.

**Table 5:** Body weight, hematological and non-hematological data follow-up for patients taking chemotherapy plus *A. cinnamomea* (AC) or placebo

	Chemotherapy + AC (*n* = 17)		*p*-value^a^	Chemotherapy + placebo (*n* = 20)		*p*-value^a^	*p*-value^b^
	Pre-test	Post-test		Pre-test	Post-test		
Hematological							
WBC (10^3^/ul)	6.1 ± 2.64	11.55 ± 1.30	0.38	5.75 ± 3.23	11.47 ± 1.48	0.34	0.10
Hemoglobin (g/dl)	11.57 ± 1.17	6.17 ± 2.77	0.43	11.75 ± 2.03	5.69 ± 2.16	0.77	0.79
Platelet (10^3^/ul)	179.11 ± 65.11	141.71 ± 63.47	<.001	201 ± 68.97	196.55 ± 74.47	0.33	0.02
Non-hematological							
Bilirubin (mg/dl)	0.64 ± 0.17	0.79 ± 0.78	0.48	0.49 ± 0.19	0.62 ± 0.28	0.12	0.62
AST (u/l)	37.88 ± 22.01	39.62 ± 26.38	0.33	27.75 ± 11.68	26.50 ± 9.73	0.30	0.07
ALT (u/l)	29.70 ± 17.50	28.70 ± 15.46	0.14	20.33 ± 10.28	29.89 ± 9.46	0.42	0.09
Creatinine (mg/dl)	0.82 ± 0.29	0.86 ± 0.30	0.11	0.72 ± .17	0.78 ± 0.17	0.10	0.73
Body weight (kg)	61.98 ± 9.09	61.24 ± 9.13	0.21	60.36 ± 11.11	59.45 ± 11.36	0.13	0.53

^a^Wilcoxon signed-rank test (Within group)

^b^Mann-Whitney *U* test (Differences in efficacy between two groups

Various treatment-related adverse events (any grade) due to the chemotherapy plus either *A. cinnamomea* or placebo in this study are reported in Table 6. Gastrointestinal complaints were the most common AEs reported during the study. Most of the gastrointestinal complaints were of grade 1–2 in the AC group. Additionally, one patient receiving placebo reported grade 3 diarrhea (increasing to ≥7 stools per day). Four patients suffered severe abdominal pain in the placebo group due to disease progression. The grade 3–4 AEs in other patients in both groups were related to disease progression, except that an 81 y/o patient with gastric cancer in the AC group developed upper gastrointestinal (GI) bleeding after 10 doses of AC.

**Table 6:** Common adverse events (AEs) of 37 cancer patients treated with chemotherapy plus either *A. cinnamomea* or placebo

	Chemotherapy + AC (*n* = 17)		Chemotherapy + placebo (*n* = 20)	
	Grade 1–2, *N* (%)	Grade 3–4, *N* (%)	Grade 1–2, *N* (%)	Grade 3–4, *N* (%)
Abdominal pain	5 (29.4)		1 (5)	4 (20)
Diarrhea	3 (17.6)			1 (5)
Dry mouth	1 (5.9)			
Blurred vision			1 (5)	
Dyspnea		2 (11.8)		
Intracranial hemorrhage		1 (5.9)		
Upper gastrointestinal bleeding		1 (5.9)		1 (5)
Infection		1 (5.9)		2 (10)

## Discussion

This double-blind randomized clinical trial examined the impact of 30-day *A. cinnamomea* treatment on the efficacy and safety associated with chemotherapy in patients with advanced cancers. Despite the strong scientific rationale in the biotechnology industry [12, 18, 19], the combination of AC and standard chemotherapy regimens failed to improve the survival and response rates of the AC group relative to those of the placebo group in patients with advanced cancers.

A recent preclinical study demonstrated the antitumor effect of AC on non-small cell lung cancer cells both in vitro and in vivo with bioluminescence imaging and indicated that the inhibition of tumor proliferation may be related to induced apoptosis [20]. Moreover, AC has been shown to enhance the cytotoxic effect of chemotherapy on ovarian cancer and hepatoma cells [13, 21]. These results have led to AC being used widely in certain populations and kept its price high in Taiwan. However, the sweet success of herbal or folk medicine agents in animal studies and the bitter aftertaste in clinical studies have recently been a major topic of discussion in research [22]. Known experimental factors, including the animal model, target selection, recommended dose, and differences in comorbidities, influence all treatments from cellular therapy to new drug application [23]. Despite the nonexistence of a clear anti-cancer effect found in this trial, given the analyses of OS and DCR of AC treatment, a potential finding is that AC does not affect the progression of cancer when combined with chemotherapy.

Advanced or metastatic cancer is predictably associated with challenges and burdens that may lead to symptoms of fatigue, toxicities of cancer therapy, and compromised patient QoL and mortality [24, 25]. The production of reactive oxygen species (ROS) is an important contributor to these symptoms and metabolic abnormalities [26]. Although AC is used for protection against oxidative damage and elimination of fatigue [27, 28], this study did not find a significant benefit on fatigue or QoL-related outcomes in patients treated with AC. Only for quality of sleep was AC more effective than the placebo. It is known that sleep disturbances affect 30–50 % of cancer patients and contribute additional risks of depression, fatigue, heightened pain, and decrement of survival rates [29, 30]. Evidence is growing that disruptions in biological rhythmicity are also relevant to cancer. The mitotic properties of cancerous cells themselves, the treatments of cancer, the time of day of treatment administration, and possibly the quality of life of cancer patients all affect outcomes [31]. A recent study reported that poor sleep quality may be associated with increases in oxidative stress indicators and decreases in free radical scavenging anti-oxidants [32]. However, few studies to date have investigated the relationship between AC and the improvement of quality of sleep. This finding in our study, that AC may improve the quality of sleep, is worthy of further exploration, but the evidence does not support a strong recommendation. Further research is needed in order to examine the potential impacts of the integrative model of care on specific outcomes, such as chemotherapy-induced and cancer-related fatigue, as well as other QoL-related concerns.

In the minds of many patients, *A. cinnamomea* is a natural component with high antioxidant and polysaccharide contents. The lack of toxicity reported in previous studies might indicate that is safe for use as a functional food ingredient [33, 34]. However, the adverse events observed in patients treated with AC in our study were generally consistent with its known adverse event profile. A study published in China concluded that the property and flavor of AC are cold and bitter [35]. According to Traditional Chinese Medicine (TCM) theory, the barrier function of gastric mucosa is inhibited after the intake of an excessive amount of bitter and cold medicine, possibly leading to damage to the stomach [36]. If these medicines are taken for an extended period or in large doses, the decreases in prostaglandin E2 and increases in products of lipid peroxidation in gastric mucosa will produce oxidative stress and its companions, such as inflammation. Our findings showed AC to cause mild to moderate irritation in the GI tract at intake, especially in debilitated patients. The incidence of GI reactions, including abdominal pain and diarrhea, was much greater in the AC group (47.1 %) than in the placebo group. In clinical practice, the AC-associated GI reactions can be alleviated by keeping pieces of ginger (*Zingiber officinale* Roscoe) in the mouth after AC intake, for this warm and acrid medicine can restrain the cold and bitterness, according to the TCM compatibility of herbal medicines [37]. Recent studies have demonstrated that ginger may provide a protective effect against drug-induced gastric injury and have a potential ulcer-preventive ability [38, 39]. However, because the precise mechanism of the interaction of AC and ginger has yet to be elucidated, we cannot exclude the possibility that an antagonistic effect of the combined agents may play some role.

The majorities of enrolled patients in both groups had advanced adenocarcinoma and were receiving similar chemotherapy regimens. The laboratory parameters regarding hematological and non-hematological safety were comparable in both treatment groups. There was a significant decrease in platelet counts within 30 days of adjuvant AC treatment. Our data also indicated a large difference in the decline in platelet levels between the two groups in patients with lung cancer (83.5 % vs. −5.0 %) and gastric cancer (22.4 % vs. −4.9 %). However, the relationship between the decrease in platelet counts and adjuvant AC treatment was not well established in our study.

Thrombocytopenia in itself is sometimes just a paper laboratory value, and the toxicity does not affect patient safety or outcome. However, a platelet count below 10 × 10^9^/L (and maybe < 5 × 10^9^/L) often leads to an increased risk of bleeding in cancer patients and can accelerate morbidity [40, 41]. Patients exposed to chemotherapeutic agents, such as cyclophosphamide (CTX), methotrexate (MTX), 5-FU, cytarabine (Ara-C), and etoposide (VP-16), are at higher risk of experiencing thrombocytopenia due to their effects on the production of blood cell progenitors [42]. In our study, few patients received the above chemotherapeutic agents, except 5-FU for liver or colorectal cancer. The extent of thrombocytopenia was analyzed in both groups, and patients with liver or colorectal cancer exhibited no significant differences from the others. Though this observation spanned only a short period, the finding may imply an association between AC and its platelet-lowering effect in patients with lung or gastric cancer. Most research has focused on the antiplatelet activity of AC as a potential therapeutic agent for treating thromboembolic disorders [43, 44]. However, no definitive proof demonstrates that AC interferes with the processes of platelet production, destruction, and pooling. We are fully aware that our sample size was limited and that the data analysis in our study had its restrictions. However, the harmful effect of AC in the treatment of cancer is unknown to the public, especially in the case of advanced cancer patients, so the findings of this study could provide some preliminary data.

The small sample size and the recruitment of patients with different types of cancer are the most important problem of our study. For this reason, the study may have had insufficient power to detect significant differences for anti-cancer effects. We know every cancer has its own characteristics and natural course, but it is important to manage the cancer-related symptoms and adverse events of chemotherapy, especially in the advanced stage. Although disappointing results could be anticipated for cancer patients in advanced stage even with the use of plant extract. We still can observe some phenomena, including safety and physical symptoms, after AC treatment. The results should be informative for health care professionals and our patients.

This clinical study had several limitations. First, this plant extract has multiple potent mechanisms and different activity, but this study was assayed only some test and, in fact is a marginal experiments. Second, the treatment lasted only 30 days, so related changes in prognostic indicators could not be demonstrated. Longer or continued AC treatment may be required for clinically significant changes in patient well-being to be observed. Third, due to the restrictions on sample collection from advanced cancer patients, the study neither evaluated the relationship between a target cancer and AC usage nor elucidated the effects of AC treatment. Finally, AC is not yet regulated by the Food and Drug Administration. Some studies previously also reported that different extracts from *A. camphorata* showed cytotoxic activity, so it is need to determine cytotoxic dose and IC50 value before being a complementary therapy [45]. Further investigation using a more sample, distinct cancer in early stage, long-term treatment, and rigorous design will be needed to determine the efficacy of *A. cinnamomea*.

## Conclusions

*A. cinnamomea* is a popular folk medicine and has attracted great attention due to its reputation for anti-cancer activity against several types of cancer, but little information is available on its clinical application. This study is the first to report the therapeutic effects of this medical fungus in advanced cancer patients receiving standard chemotherapy. The mean 6-month survival rate was not significantly different between groups (5.4 months vs. 5 months, AC versus placebo, respectively). The only significant difference between groups was the platelet counts, which were lower in the AC group, and sleep quality, which was significantly improved in the AC group. Overall, AC combined with chemotherapy did not improve the outcomes in the advanced cancer patients.

## Abbreviations

5-FU: 5-fluorouracil
AC: *Antrodia cinnamomea*
AE: Adverse event
Ara-C: Cytarabine
CAM: Complementary and alternative medicine
CR: Complete response
CTCAE: National Cancer Institute’s Common Terminology Criteria for Adverse Events
CTX: Cyclophosphamide
DCR: Disease control rate
ECOG: Eastern co-operative oncology group
EORTC-QLQ-C30: European organization for research and treatment-quality of life questionnaire
GI: Gastrointestinal
KCGMH: Kaohsiung Chang Gung Medical Hospital
MDR: Multi-drug resistance
MFI: Multidimensional fatigue inventory
MTX: Methotrexate
NCCN: National Comprehensive Cancer Network
NOAEL: No-observed-adverse-effect-level
OS: Overall survival
p-AKT: phospho-AKT
PR: Partial response
PS: Performance status
QoL: Quality of life
RECIST: Response evaluation criteria in solid tumors
ROS: Reactive oxygen species
SD: Sprague-dawley
SD: Standard deviation
SD: Stable disease
SNQ: Taiwan Symbol of National Quality
TCM: Traditional chinese medicine
VP-16: Etoposide
